# In Situ Nanostress Visualization Method to Reveal the Micromechanical Mechanism of Nanocomposites by Atomic Force Microscopy

**DOI:** 10.1021/acsami.2c22971

**Published:** 2023-02-28

**Authors:** Xiaobin Liang, Takashi Kojima, Makiko Ito, Naoya Amino, Haonan Liu, Masataka Koishi, Ken Nakajima

**Affiliations:** † Department of Chemical Science and Engineering, School of Materials and Chemical Technology, Tokyo Institute of Technology, Ookayama 2-12-1, Meguro-ku, Tokyo 152-8550, Japan; ‡ AI Laboratory, The Yokohama Rubber Co., Ltd., 2-1, Oiwake, Hiratsuka, Kanagawa 254-8601, Japan

**Keywords:** nanostress, visualization, in situ, nanocomposites, atomic force microscopy, mechanism

## Abstract

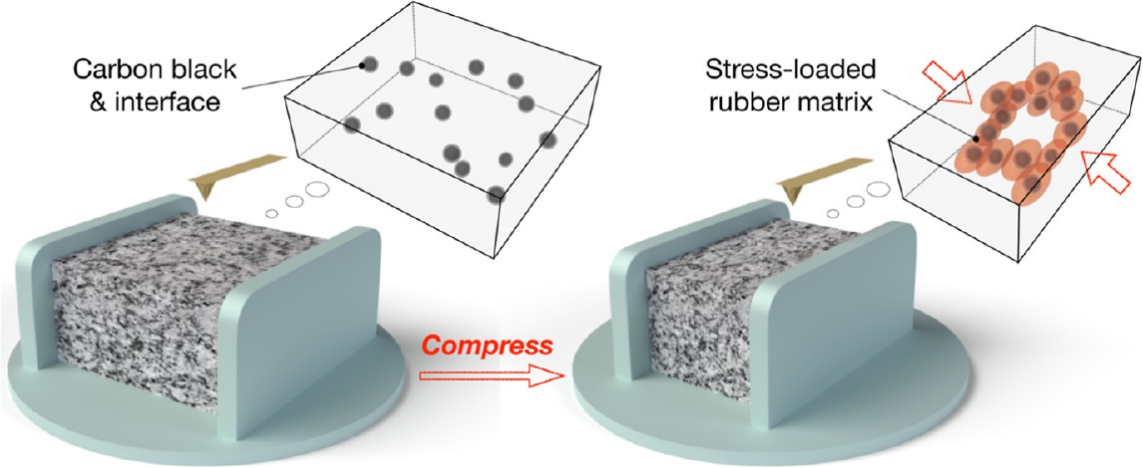

An in situ atomic force microscopy (AFM) nanomechanical technique was used to directly visualize the micromechanical behaviors of polymer nanocomposites during compressive strain. We obtained a stress distribution image of carbon black (CB)-filled rubber at the nanoscale for the first time, and we traced the microscopic deformation behaviors of CB particles. Through this experiment, we directly revealed the microscopic reinforcement mechanisms of rubber composites. We found that CB-filled rubbers exhibited heterogeneous local microscopic deformations, which were related to the dispersion of CB particles in rubber matrices. The local stress distributions of the rubber composites showed heterogeneity, and the stresses were concentrated in the regions near the CB particles during compression. The area of stress concentration gradually expanded with increasing strain and eventually formed a stress network structure. This stress network bore most of the macroscopic stress and was considered the key reinforcement mechanism of CB-filled rubber. The stress transfer process in the rubber matrix was visualized in real space for the first time. Based on the image data from the AFM experiments, we used finite-element method (FEM) simulations to reproduce the microscopic deformation process of CB-filled rubber. The stress distribution images simulated by FEM showed heterogeneity consistent with AFM. In this study, an in situ visualization of material deformation confirmed the predictions of microscopic deformation behavior from previous theories and models; it also provided new insights into the microscopic reinforcement mechanisms of CB-filled rubber composites based on microscopic stress distribution images.

## Introduction

Polymer nanocomposites (PNCs) are widely used for reinforcing and modifying polymers to meet the present demands for superior material performance and low carbon emissions.^1−4^ Nanoparticle-filled rubber is a typical PNC with substantially better mechanical properties than those of original rubbers.^5−7^ The most prevalent filler type in the rubber industry is carbon black (CB), which has been used for over 100 years to improve the elastic modulus, fracture strength, and wear resistance of rubber in a simple and significant manner.^8,9^ Over the past few decades, the use of silica in tire rubber has attracted considerable attention,^10,11^ while nanofillers with special geometries, such as carbon nanotubes and graphene, have also been extensively studied.^12−15^ Although various nanofillers have been applied to rubber, it is still crucial to understand the mechanisms of CB-filled rubber to improve and design other types of PNCs. Over the past decade, advanced characterization tools and instruments have brought extensive new information to help us better understand the filler reinforcement process.^16−18^ In particular, high-resolution microscopic imaging techniques have made it possible to visualize materials at the nanoscale, such as atomic force microscopy (AFM),^19,20^ scanning electron microscopy,^21,22^ and transmission electron microscopy.^23−25^ Among them, AFM not only provides nanoscale morphological information about material surfaces but also visualizes nanomechanical properties; thus, it is widely used for researching rubber.^26,27^ AFM can evaluate the thickness of the bound rubber layer between the filler and the rubber and investigate the effects of fillers on the physical properties of rubber matrices through nanoelastic modulus mappings.^28,29^ In addition, a nanorheological AFM technique provides a new approach for studying the viscoelastic behaviors of rubber samples by measuring the nanoscale storage and loss moduli through the contact oscillation of a probe on the material surface.^30^

Although advanced imaging techniques have become powerful tools for PNC research,^31,32^ microscopic visualization techniques for observing material deformation behaviors and stress distributions are still lacking. In recent years, the application of AFM nanomechanics for measuring deformed samples has made it possible to trace the microscopic deformation behavior of materials.^33,34^ For example, Morozov used AFM nanomechanics to study the surface morphology and micromechanical properties of filled rubber in tension and found that transverse nanocracks appeared near fracture and that the crack mechanism depended on the filler concentration and the distance from the crack tip.^35^ Recently, we developed an AFM-based nanoscale visualization method to quantitatively measure the microscopic stresses of CB-filled isoprene rubber (IR) in deformation; we proposed a model to predict the macroscopic tensile stresses based on the microscopic stress distributions and microscopic spatial structures.^36^ However, this method measures different samples of the same composition at different strains, so it is not possible to trace the deformation process in the same region. We can only establish the correlation between macroscopic stress and microscopic stress through simple series and parallel models. The lack of direct observation of the stress development with strain limits our understanding of the mechanical mechanisms of composites. Therefore, in this study, we improved the experimental methods and performed in situ observations on the microscopic strain and stress distributions of CB-filled IR at the nanoscale in situ under controlled compressive strain; this process is called in situ AFM nanomechanics. For the first time in experiments, the in situ microscopic stress distributions of polymer composites were measured directly during deformation, which intuitively revealed the microscopic deformation behaviors and reinforcement mechanisms of rubber composites. In addition, this study reproduces the microscopic deformation behavior of the material through finite-element method (FEM) simulation based on the microscopic data of AFM. This method will allow us to truly observe the deformation behavior and stress occurrence of materials directly at the nanoscale, providing a reference for the study of mechanical mechanisms of materials and the design of new materials.

## Results and Discussion

### Heterogeneity of Microscopic Deformation

**Figure 1:**
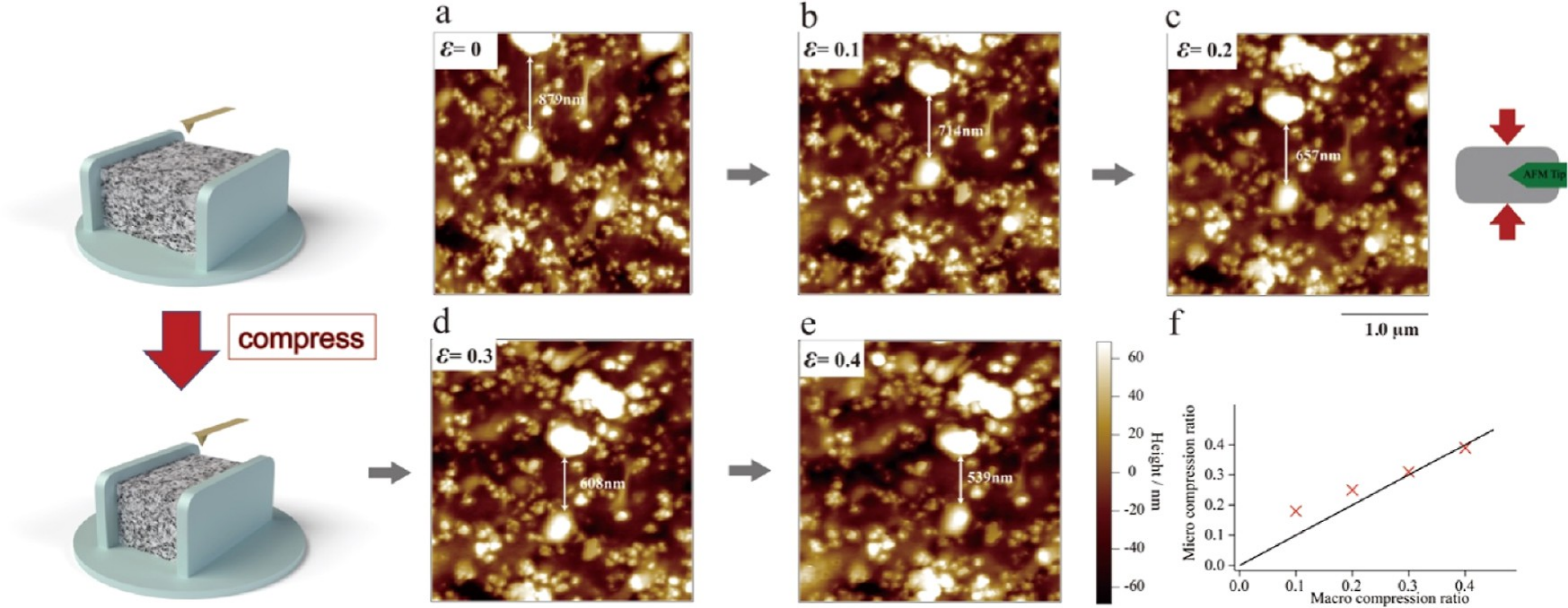
(a–e) In situ AFM height images of CB-filled IR at macroscopic compressive strains ε = 0, 0.1, 0.2, 0.3, and 0.4. The scan size is 3.0 μm. (f) Comparison of the microscopic strain between the two CB aggregates in (a–e) and the applied macroscopic strain.

In this section, we focus on the displacements of CB particles during deformation to elucidate the microscopic deformation mechanisms of 40 phr CB-filled IR samples. Figure 1a–e shows in situ height images of CB-filled IR at different compressive strains ε (there is a slight drift of the image position due to AFM instability). The white areas in Figure 1 are CB particles or CB particle aggregates, the sizes of which range from a few tens to hundreds of nanometers. Most of the CB particles are inhomogeneously distributed in the IR matrix as aggregates. The shrinkage trend of the CB particles along the compression direction (arrow direction) is observed with increasing macroscopic compressive strain ε. The in situ microscopic deformation behaviors under compressive strains can be tracked through the displacements of CB particles. In addition, we did not observe a significant change in surface height with strain, so the CB particles buried under the surface were considered to have no significant vertical displacement in the range of compressive strains less than 0.4, i.e., they did not affect the observed surface. In this study, we focus on the displacement changes in the two largest CB aggregates shown in Figure 1a. The displacement of the CB aggregate is 879 nm in the undeformed state, and it decreases to 539 nm when the strain gradually increases to ε = 0.4, as shown in Figure 1e. Based on the changes in the microscopic displacement of CB particles, we can calculate the microscopic strain ε_micro_ between two CB aggregates as follows
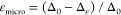
1where ε_micro_ is the microscopic strain, Δ_0_ is the initial distance between the two CB aggregates, and Δ_ε_ is the distance between the two CB aggregates at the macroscopic strain ε. A comparison between the two CB aggregates (marked in Figure 1a–e.) and the applied macroscopic strain is shown in Figure 1f. If the material is completely homogeneously deformed during compression, the AFM data should precisely coincide with the black line, i.e., the microscopic strain is exactly the same as the macroscopic strain. However, we find that the microscopic deformation deviates from the macroscopic strain at ε = 0.1 and 0.2 and is instead consistent with the macroscopic strain at ε = 0.3 and 0.4. This observation indicates that the local deformation of the CB/IR sample is heterogeneous, and even the same microscopic regions exhibit different deformation behaviors under different strains. This heterogeneous deformation mechanism of filled rubber has been reported in several studies^16,37^ and is widely used in theories and models to describe the deformation behaviors of filled rubbers.

**Figure 2:**
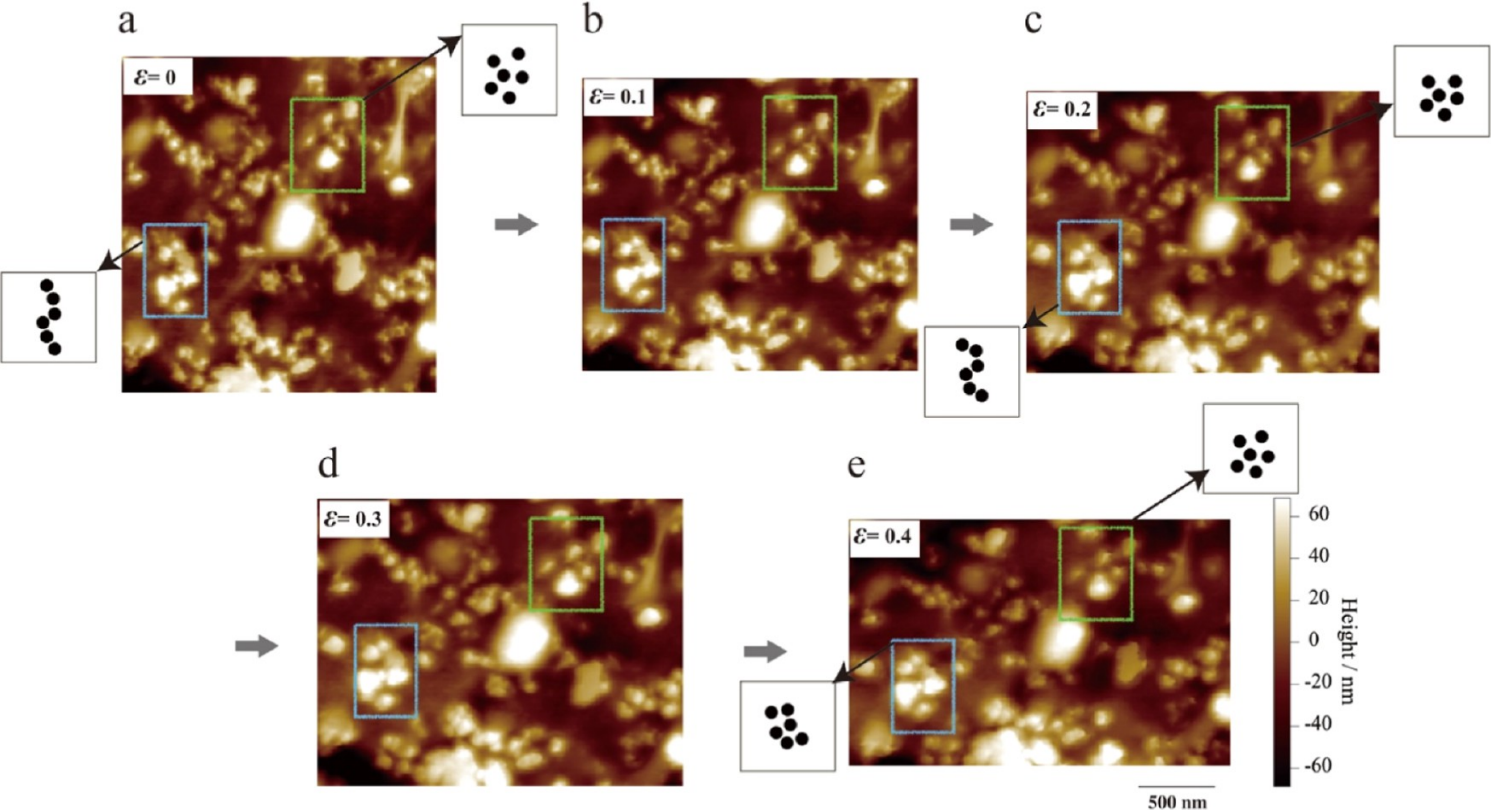
(a–e) In situ AFM height images of the same region at macroscopic compressive strains ε = 0, 0.1, 0.2, 0.3, and 0.4 derived from the partial region expansion in Figure 1a–e.

The microscopic deformation mechanisms of the filled rubbers are complex and exhibit differences under different strains. To elucidate the mechanisms, we concentrate on tracking the deformations of small regions in situ; we expand part of the region shown in Figure 1a–e to the one shown in Figure 2a–e. Here, we focus on two selected areas in Figure 2 (green and blue boxes). First, the CB particles in the green region are uniformly dispersed, and this local network structure allows the CB particles to be more firmly positioned without showing the very large shrinkage deformation that occurs due to compression. Some filled rubber mechanics model studies^38,39^ suggest that the bound rubber layer between the CB and the rubber matrix results in the part of the rubber matrix being restrained near the overlapping region, thus enhancing the reinforcement effect. Most of this restricted rubber structure appears when the filler is closely distributed and is considered to be difficult to deform. However, considering that the thickness of the binding rubber layer is only ∼10 nm, the overlap of the bound rubber layer in the green box region is difficult to achieve because the distance between the CB particles is approximately 50–65 nm. Therefore, we speculate that even if the bound rubber layers are not in direct contact, a similar restricted rubber effect occurs during deformation. The microscopic stresses between particles are crucial for this mechanism, which is discussed in the next section.

In the blue region, the CB particles are not uniformly dispersed and are instead distributed in the rubber matrix in a chain shape. Since the CB particles are far apart, they cannot support each other to form a local network structure during deformation. As a result, a large shrinkage deformation occurs under compressive strain, and finally, a stable structure with a uniform particle distribution is formed. In situ AFM images provide a visualization of the deformation behavior of the filled rubber at the nanoscale and show that the dispersion state of the filler influences the local microscopic deformation, i.e., the uniformly dispersed regions are more difficult to deform, and the unevenly dispersed regions are more prone to deformation.

### Heterogeneity of Microscopic Stress Distributions

**Figure 3:**
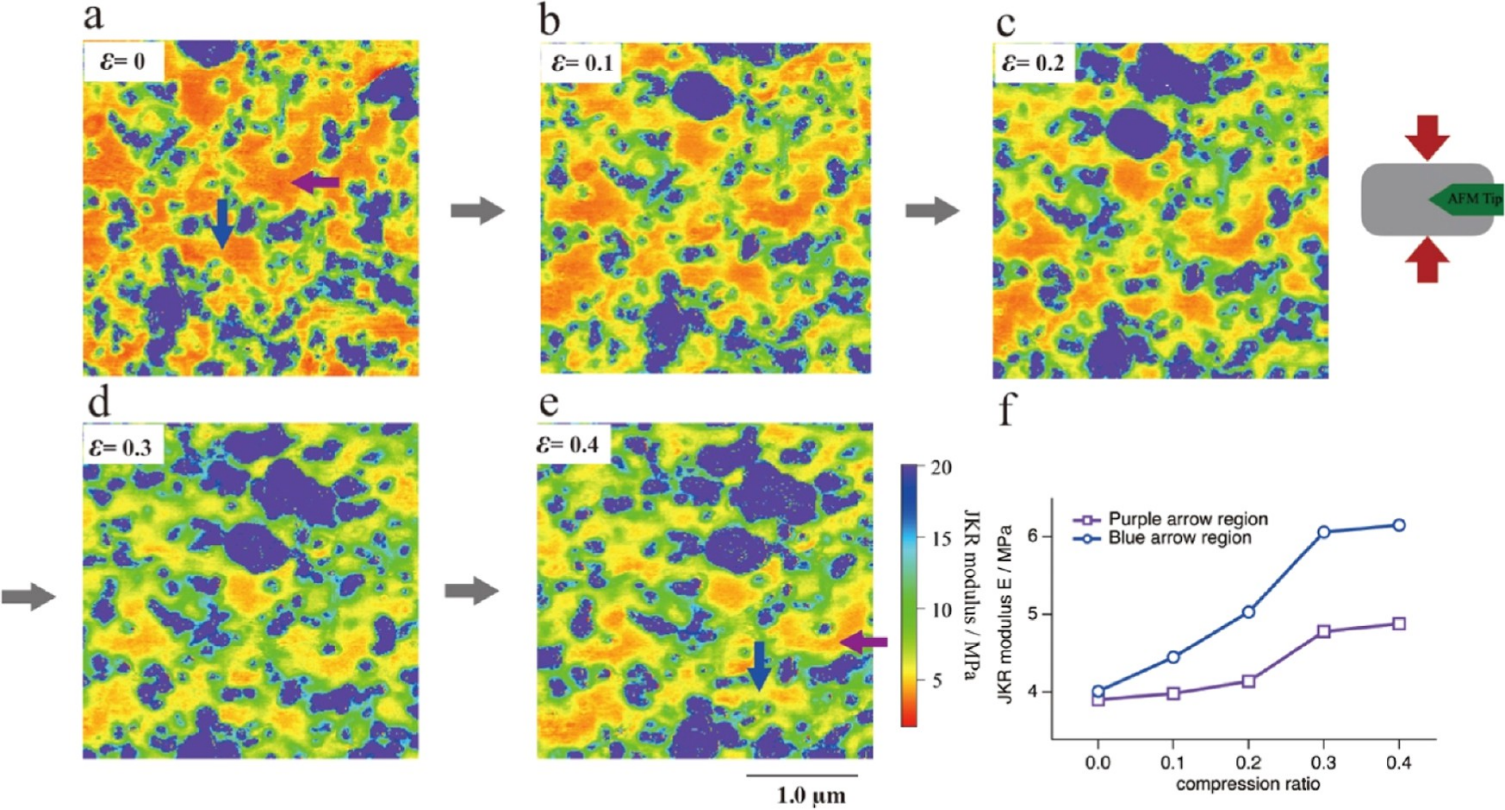
(a–e) In situ AFM JKR modulus images of CB-filled IR at macroscopic compressive strains ε = 0, 0.1, 0.2, 0.3, and 0.4. The scan size is 3.0 μm. (f) Variation in the JKR modulus with strain for two specific regions.

AFM is a powerful technique for characterizing the morphologies and structures of materials at high resolution, and it provides information on the nanomechanics of materials. Figure 3 shows the Johnson–Kendall–Roberts (JKR) modulus images at different compressive strains. The undeformed JKR modulus image (Figure 3a) shows the CB phase with a high modulus (blue–green), the IR rubber matrix with a low modulus (orange), and the interface region (yellow) between the two phases. In this figure, since the CB filling amount of 40 phr is close to the percolation threshold, the apparent continuous network structure is not observed in the two-dimensional AFM images. The modulus and the interfacial region of the rubber matrix show an overall increasing trend with increasing compressive strain (see Figure 3b–e). This increase in modulus is due to the stress distribution of the deformed sample, which has been reported in our previous studies.^36,40^ In addition, during compressive strain, the modulus of the rubber matrix near the interface region increases significantly, connecting the CB particles to each other and forming a stress-bearing network structure. This network structure is a key mechanism for nanoparticle-reinforced rubber, which we will discuss in detail later. Notably, the rubber matrix away from the interface exhibits a slight increase in the modulus due to the partial stress-bearing behavior. Trabelsi et al.^41^ suggested that high local stresses in rubber near the filler are based on the stretch-induced crystallization behavior in filled natural rubber, while the local stresses in rubber distant from the filler are the same as those in unfilled rubber. In this study, in situ AFM has proven this mechanical behavior for the first time in real space. Interestingly, this modulus increase shows inhomogeneity. As shown in Figure 3a,e, the blue and purple arrows show almost the same modulus when undeformed, while the blue arrow region shows a much higher increase in modulus than the purple arrow region when the macroscopic strain ε = 0.4. Figure 3f shows the JKR modulus trends in both regions, and it is obvious that the increase in the modulus is higher in the purple arrowed region than in the blue arrowed region, especially at high compressive strains. Similar to the inhomogeneous microscopic deformation mechanism, the microscopic stress distribution exhibits heterogeneity.

**Figure 4:**
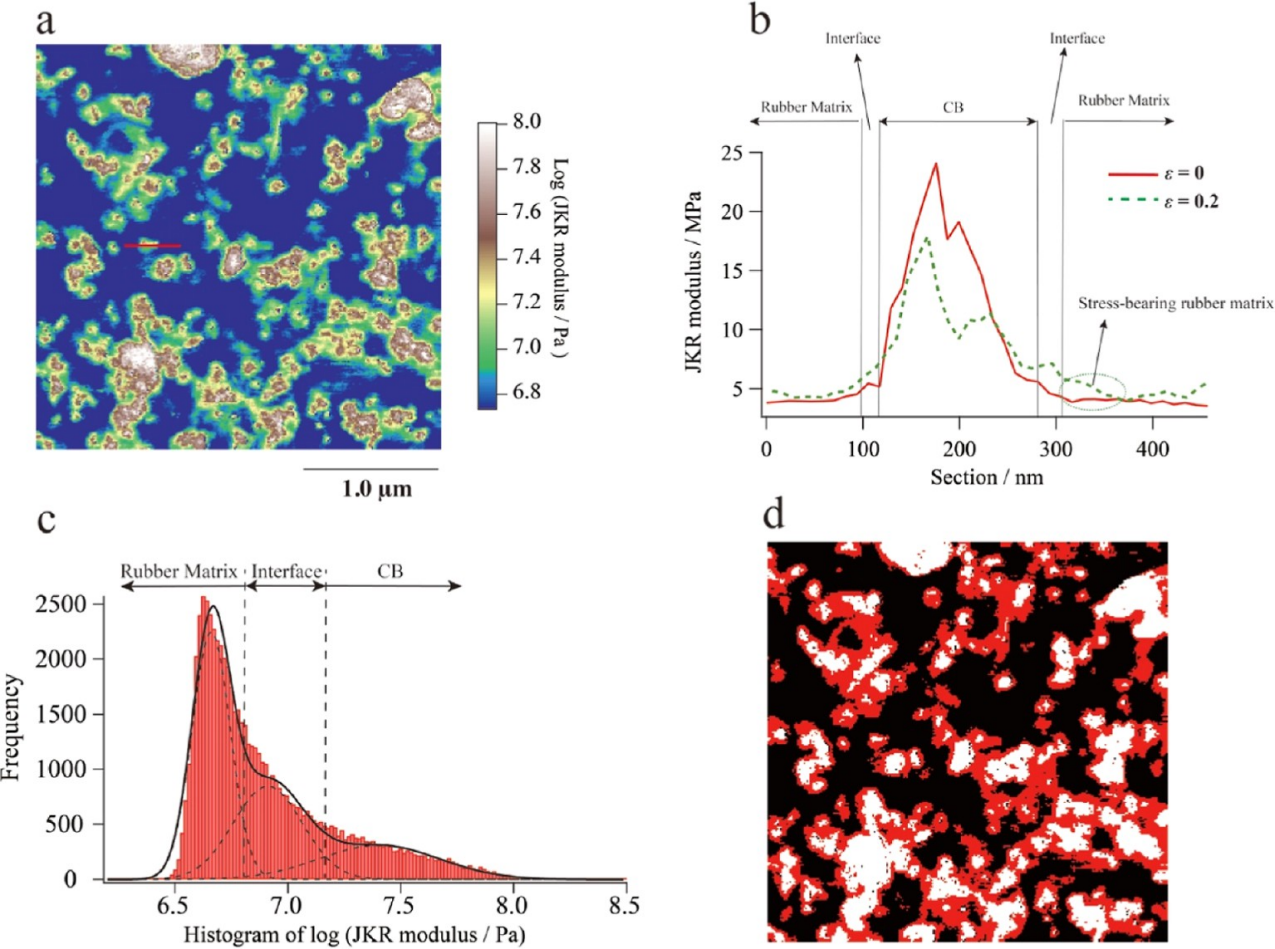
(a) Logarithmic image of the JKR modulus for CB-filled IR under undeformed conditions. (b) JKR modulus section (red line) at the red line in(a) when undeformed and the JKR modulus section (green dashed line) at the same position at a compressive strain of 0.2. (c) Histogram of the JKR modulus, which is described well by three independent Gaussian functions. (d) Ternary image of the JKR modulus for 40 phr CB-filled IR based on the separation of (b).

To further elucidate the mechanism of inhomogeneous stress distribution in filled rubber, it is necessary to quantitatively characterize and discuss the contents and moduli of each phase. Figure 4a shows the logarithmic image of the JKR modulus for CB-filled IR when undeformed. The JKR modulus cross section of the red line in Figure 4a is shown as the red line in Figure 4b, and the modulus of the CB phase is ∼60 MPa, which is much lower than the typical modulus value of CB (∼10 GPa).^42^ The reason for underestimation is that the hard CB particles are embedded in a soft rubber matrix, causing the pressure to only deform the rubber under the CB and not the CB itself; therefore, the CB modulus is not discussed in this study.^43^ Therefore, it is difficult to distinguish each phases only by the value of the JKR modulus. Fortunately, the histogram of the logarithm of its moduli (Figure 4c) is described well by three independent Gaussian functions, making it possible to quantitatively evaluate the moduli and ratios of each phase. Note that for the undeformed samples, the intermediate-modulus region contains only the interface, whereas for the deformed samples, the intermediate-modulus region contains the interface and the stress-bearing rubber matrix near the interface. By separating each phase, a ternary image of the CB-filled IR rubber is obtained, as shown in Figure 4d.

**Figure 5:**
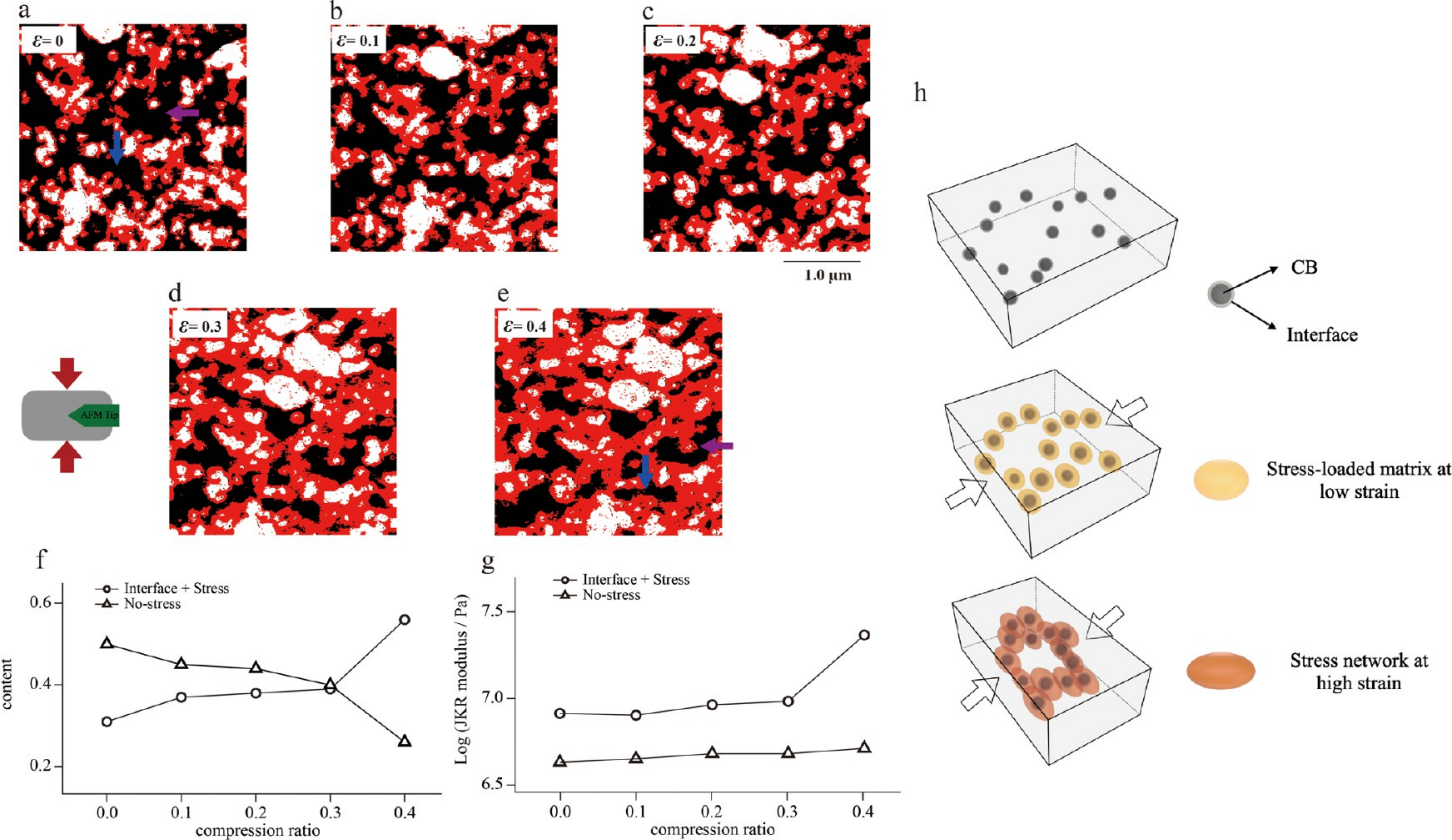
(a–e) Ternary images based on modulus separation at macroscopic compressive strains ε = 0, 0.1, 0.2, 0.3, and 0.4. (f) Ratio of the rubber matrix and intermediate-modulus region as a function of compressive strain. (g) Average modulus values of the rubber matrix and intermediate-modulus region as a function of compressive strain. (h) Schematic diagram of the deformation micromechanical mechanism.

The ternary images based on the modulus separation at different compressive strains are shown in Figure 5a–e. The white areas are the CB particles or CB particle aggregates, and their proportions remain consistent regardless of compressive strain. The black areas denote the rubber matrix, the proportion of which gradually decreases with increasing strain. The red areas denote the intermediate-modulus region, as mentioned before, which contains the interface and rubber matrix of the local stress concentration (when deformed). We find that the proportion of the stress-bearing rubber matrix (red area) gradually increases with increasing strain and connects to form a network structure called the stress network. This stress network formation process is first observed in real space by in situ AFM, which plays a stress-bearing role when strain occurs in filled rubber. To gain insights into the mechanical mechanisms of the stress network, we focus on the heterogeneous microscopic stress distribution in the rubber matrix shown in Figure 5a with blue- and purple-arrowed regions (specific regions are the same as in Figure 3a,e). The purple arrow area is gradually surrounded by the stress network during the compression process, forming a structure similar to the occluded rubber structure; thus, this area is difficult to deform and can bear less stress than the nonoccluded region. On the other hand, the matrix of the blue arrow is in a relatively open structure, resulting in greater deformation during compression, which in turn bears more stress than the occluded rubber region. The obstructed rubber structure has been proposed in many theories and models,^44,45^ and it is generally believed to increase the effective volume of the filler during strain. The experimental results of in situ AFM not only show that the obstructed rubber structure is formed by the filler and bound rubber layer surroundings but also show that the stress network surrounding the matrix during deformation produces the mechanical mechanisms of the obstructed rubber.

Quantitative analyses of micromechanical properties show similar conclusions. As shown in Figure 5f, the ratio of the intermediate-modulus region increases from 31% (interface only) to 56%, and the ratio of the rubber matrix decreases to approximately 24% as the compressive strain increases from 0 to 0.4. The intermediate-modulus region gradually increases with strain, as shown in the JKR modulus histogram in Figure S1. The green dashed line in Figure 4b shows the JKR modulus of the rubber around the CB particle at strain ε = 0.2, and the comparison with the undeformed modulus (red line) shows a significant increase in the modulus of the rubber matrix near the interface. This intermediate-modulus appears in the region near the interface and gradually spreads to the rubber matrix with strain. At the compressive strain ε = 0.4, the ratio of the intermediate-modulus region exceeds 50%, which transforms into the “matrix phase”, thus forming a stress network. In the modulus–strain curve (Figure 5g), there is only a small increase in the modulus of the rubber matrix, which indicates that the matrix bears only a small amount of the stress under compressive strain, which is consistent with the previous suggestion. Interestingly, the modulus values in the medium-modulus region do not increase significantly from strains ε = 0 to 0.3, where the rate of increase is close to that of the rubber matrix. The stress effect is mainly manifested in the expansion of the stress concentration area. When the strain is increased to 0.4, the modulus in the intermediate region increases significantly, and the stress network begins to bear most of the stress. Therefore, we propose a micromechanical mechanism (Figure 5h) as follows: at the initial stage of strain, the matrix near the CB bears stress and forms an intermediate-modulus region, the proportion of which gradually increases with increasing strain, eventually forming a stress network. After the stress network is formed, the network bears most of the stresses, which leads to a significant increase in the modulus value of the network.

### Comparison of FEM Simulation and AFM Measurements

**Figure 6:**
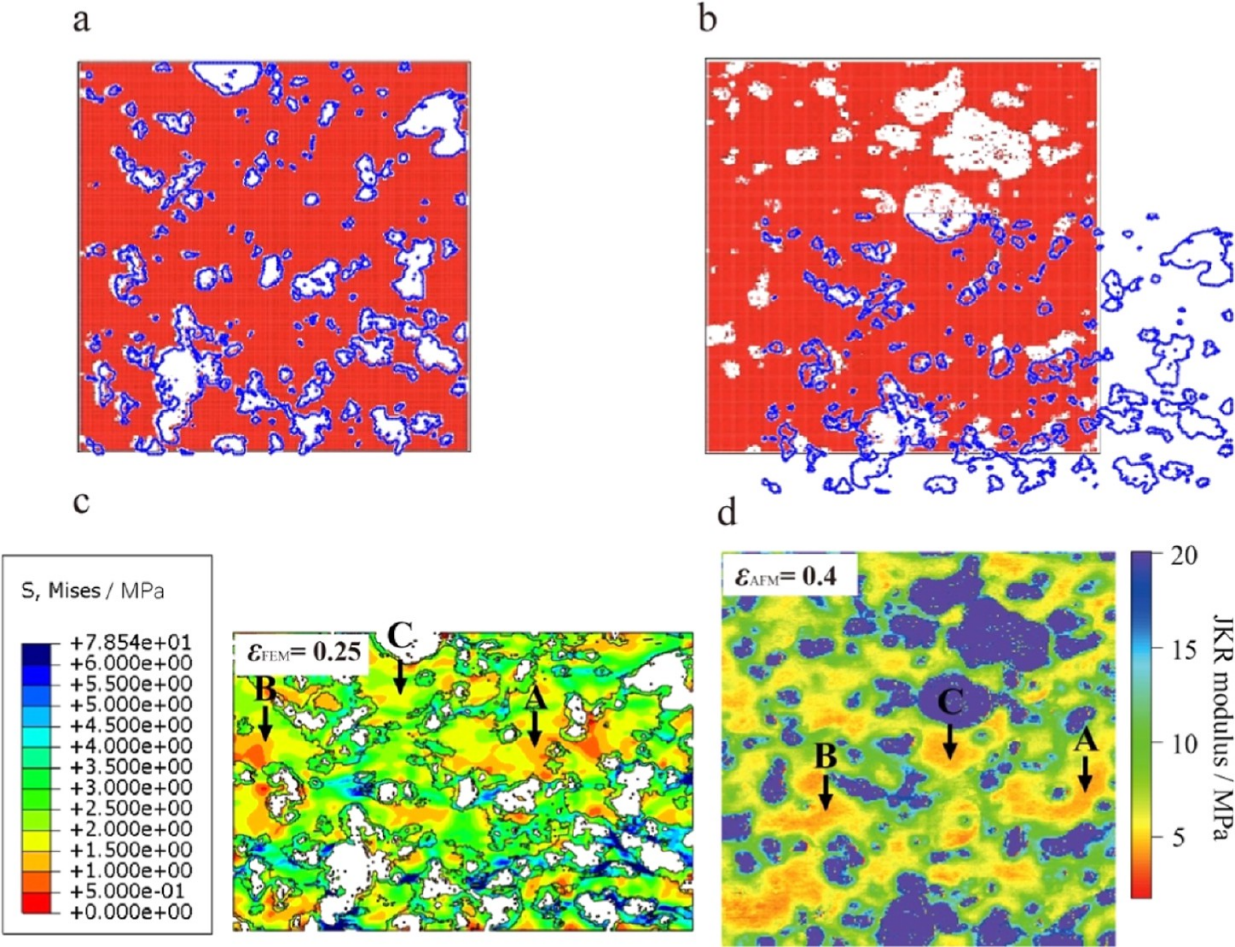
(a) Initial image of the FEM (blue) based on the AFM binarized image (red and white). (b) AFM binarized image at ε = 0.4 and FEM image at ε = 0.25. (c) von Mises stress distribution of FEM at ε = 0.25. (d) AFM JKR modulus image at ε = 0.4.

Figure 6 shows a comparison between the AFM experimental results and the FEM simulation images. The FEM simulation created the initial nanoparticle-filled rubber structure based on the AFM image data (see Figure 6a) and defined the nanoparticle modulus as 100 MPa, the rubber matrix modulus as 4 MPa, and the interfacial region modulus as 10 MPa based on AFM nanomechanical measurements. By applying a compressive strain in the vertical direction, the structural images and stress distributions at different compressive strains are obtained. A comparison of the structural images of AFM and FEM at different strains is shown in Figure S2, where both remain in good agreement at low strains. However, at high strains, the FEM shows a larger deviation from AFM because the FEM uses a 2D plane strain model for its calculations, which results in more constraints during deformation than the actual sample (3D structure) in the AFM measurements. Therefore, in this study, we ignore the difference in strain and focus on the more important information about the microscopic stress distribution. Figure 6b shows a superimposed comparison of an AFM image with strain ε = 0.4 and the FEM image with strain ε = 0.25, where the red and white images are AFM and the blue image is FEM; the results basically match, which indicates that FEM can effectively model the microscopic compression deformation behavior. This finding indicates that the microscopic deformation behavior is influenced by the microstructures and moduli of each phase and can be predicted based on reliable AFM experiments. However, there is a difference in the strain ε between the AFM and FEM models. Figure 6c shows the von Mises stress distribution at a strain of ε = 0.25, which exhibits inhomogeneities similar to those of the AFM modulus plot (Figure 6d). It is important to note that the FEM simulates the stress distribution, which is different from the JKR modulus of AFM. Therefore, we focus on the qualitative relationship between the change in the JKR modulus and the simulated stresses. In the simulated images, obvious stress concentration regions appear near the CB particles and connect to form a network structure. Moreover, the regions of low stress distributions in the simulations (Figure 6c, regions A, B, and C) match the regions of low moduli in the AFM images (Figure 6d, regions A, B, and C). This observation again confirms our suggested microscopic deformation mechanisms of the filled rubber samples. In situ AFM can provide accurate microscopic information for theoretical models and mechanical simulations through in situ observation of the microscopic deformation behaviors of PNC materials.

## Conclusions

In this study, we used a compressible sample holder to visualize the microscopic deformation and microscopic stress distribution of CB-filled IR composites under controlled compression based on in situ AFM nanomechanical analyses. The microscopic deformation mechanisms of rubber composites are revealed by tracking the displacement changes in local CB particles during deformation. The local microscopic deformation depends on the spatial dispersion of the CB particles, where the uniformly dispersed regions are more difficult to deform, and the unevenly dispersed regions are more easily deformed to form a stable CB dispersion structure. Additionally, the results of the in situ microscopic stress distribution reveal an interesting stress transfer process: at low strains, the stress is mainly distributed in the region around the CB and shows a tendency to expand with increasing strain; when the compressive strain is ε = 0.3, the stress concentration areas are connected and form a stress network structure; when the strain further increases, the stress network mainly bears the macroscopic stress so that the rubber composite material can withstand greater stresses to achieve the reinforcement effect. Based on the structural and modulus data from AFM, we simulate the compression process of rubber composites using FEM. The two techniques show similar stress distribution inhomogeneities. This finding indicates that in situ AFM can directly provide not only visualizations of the microscopic deformation behaviors of materials but also accurate microscopic information for theoretical and simulation studies. It is expected that the method can not only be applied to PNC but also play an important role in the study of mechanical mechanisms of biological materials, hydrogels, and other materials.

## Methods

### Materials and Sample Preparation

The material used in this study was a rubber composite with 100 phr of IR and 40 phr of high-abrasion furnace grade CB (N330, mean particle size of 84 nm); the detailed composition of this material is shown in Table 1. All raw materials were commercially available. To obtain a smooth material surface for AFM imaging, the samples were ultramicrotomed at −120 °C using a Leica EM FC6 (Leica Microsystems GmbH Wetzlar, Germany), with a cutting direction perpendicular to the compression direction. AFM measurements under controlled compression were then performed by using a specially designed sample holder.

**Table 1:** Formulation of the CB-Filled Isoprene Rubber

component	composition (phr)
IR	100
sulfur	2.0
stearic acid	1.0
zinc oxide	5.0
*N*-cyclohexylbenzothiazole-2-sulfenamide	1.0
CB	40

### AFM Measurements

The AFM measurements were conducted using a Nanoscope V with MultiMode 8 in the PeakForce QNM mode (Bruker AXS, USA). The samples were scanned at a peak tapping force of approximately 2 nN using rectangular silicon cantilevers with nominal spring constants of 0.51 N/m (OMCL-TR800PSA, Olympus Micro Cantilevers). The actual spring constants were measured by the thermal tuning method. The oscillation frequency of the Z-piezo was 1.0 kHz, and the peak-force amplitude was 250 nm. The force curves were collected over selected 3.0 μm surface areas at resolutions of 256 pixels × 256 pixels. Considering the high adhesion and low modulus of rubber, its Tabor parameter is approximately 290, so the force curves were analyzed in this study using the JKR contact model.^46,47^ The Young’s modulus *E* and bonding energy *w* were represented by the following two equations based on the JKR model
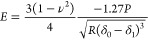
2
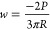
3where ν is Poisson’s ratio, *R* is the radius of curvature for the probe tip, δ is the sample deformation, and *P* (<0) is the maximum adhesive force, the schematic diagram and experimental data of which are shown in Figure S3. In addition, the tip probe is assumed to be a sphere with radius *R* in the calculation; please refer to Figure S4 for details.

### FEM Simulation

Abaqus, commercial FEM software, was used for the FEM simulation. Based on the sample binarized image (256 × 256 pixels), a two-dimensional simulation model with two material phases—polymer and filler—was created. The simulation model had a total of 65,536 quadrangular plane strain elements of 256 × 256 pixels, and the material models of the polymer and filler phases were assumed to be neo-Hook material models. Young’s modulus and Poisson’s ratio were assumed to be, respectively, 4 MPa and 0.49 for the polymer phase and 100 MPa and 0.3 for the filler phase. The moduli were defined based on AFM nanomechanical measurements. In addition, each boundary line of the simulation model was specified to be straight, and the compression deformation value was found from the prescribed displacement, please refer to Figure S5 for the detailed boundary conditions.

## Supporting Information Available

JKR modulus histogram; microstructural comparison of AFM and FEM; typical force–deformation curves; and comparison of the probe shape (PDF)
