# Supplementary material for: In Situ Nanostress Visualization Method to Reveal the Micromechanical Mechanism of Nanocomposites by Atomic Force Microscopy
Source: ACS Appl Mater Interfaces. 2023 Feb 28;15(9):12414–22. doi: 10.1021/acsami.2c22971 (PMC9999342; doi:10.1021/acsami.2c22971)
Supplement: Supplementary file 1 — am2c22971_si_001.pdf [file am2c22971_si_001.pdf]

Supporting Information

An in Situ Nanostress Visualization Method to Reveal the Micromechanical Mechanism of Nanocomposites by Atomic Force Microscopy

*Xiaobin Liang[1], Takashi Kojima[2], Makiko Ito[1], Naoya Amino[2], Haonan Liu[1], Masataka Koishi[2], Ken Nakajima[1]**

[1]Department of Chemical Science and Engineering, School of Materials and Chemical Technology, Tokyo Institute of Technology, Ookayama 2-12-1, Meguro-ku, Tokyo 152-8550, Japan

[2]AI Laboratory, The Yokohama Rubber Co., Ltd., 2-1, Oiwake, Hiratsuka, Kanagawa 254-8601, Japan

*Correspondence: knakaji@mac.titech.ac.jp

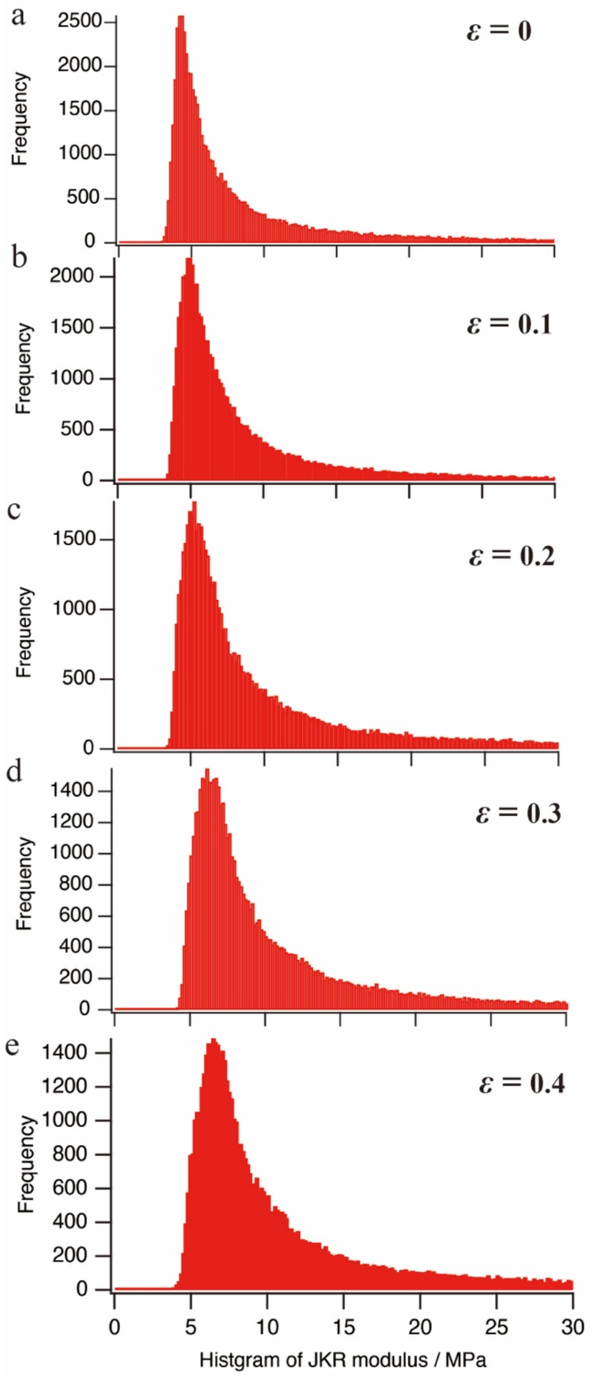

Figure S1. Comparison of the structural images from AFM experiments and FEM simulations at different strains, where the compressive strains are 0 (a), 0.1 (b), 0.2 (c) and 0.4 (e), respectively.

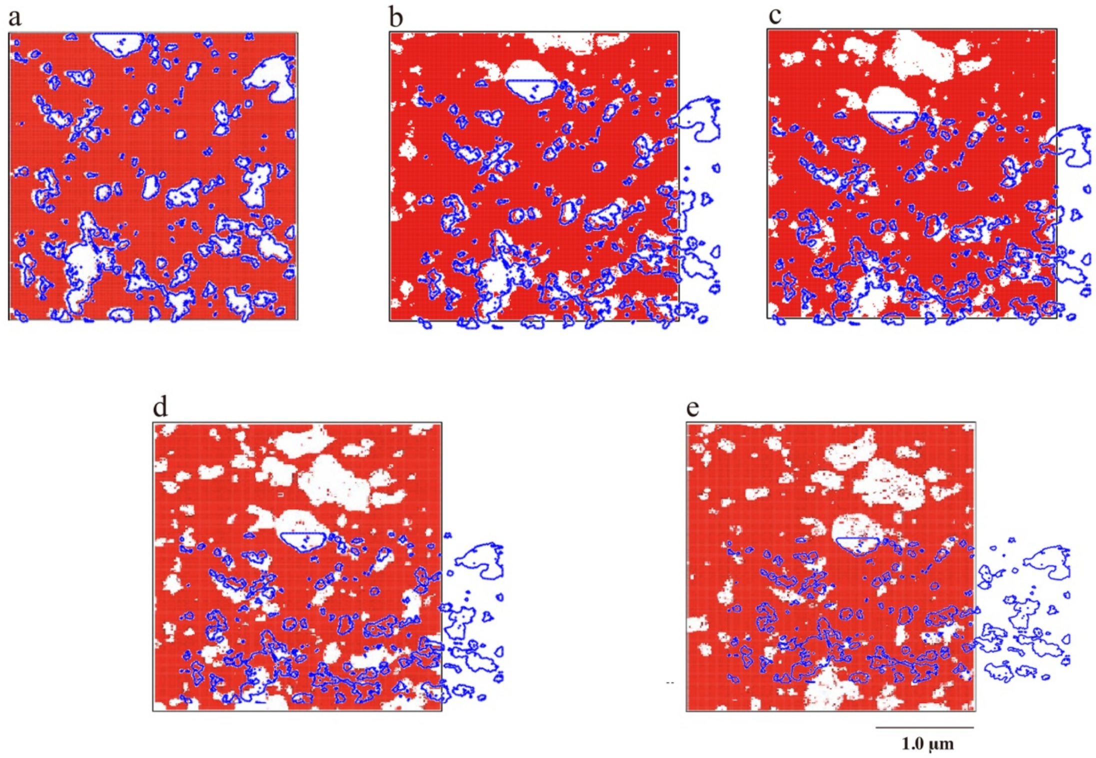

Figure S2. Comparison of the structural images from AFM experiments and FEM simulations at different strains, where the compressive strains are 0 (a), 0.1 (b), 0.2 (c) and 0.4 (e), respectively.

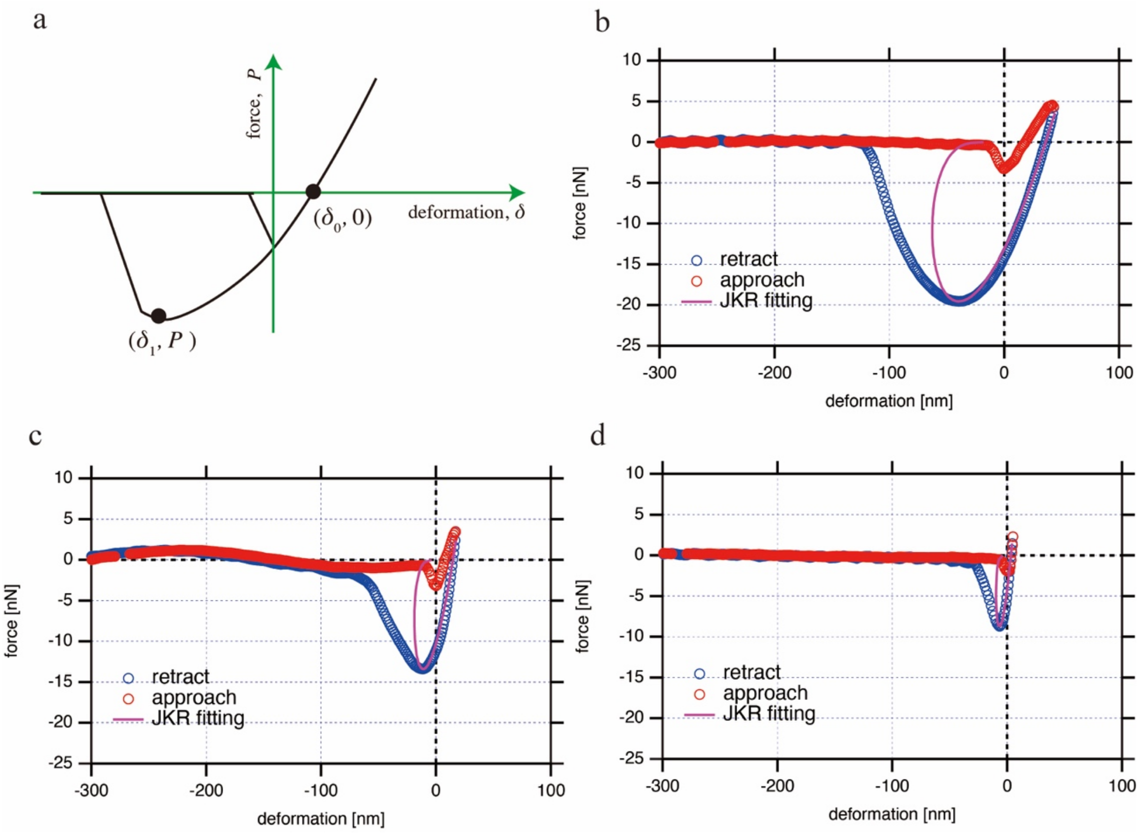

Figure S3. A schematic of force–deformation curve (a); typical force-deformation curves of the rubber (b), interface (c), and CB phases (d).

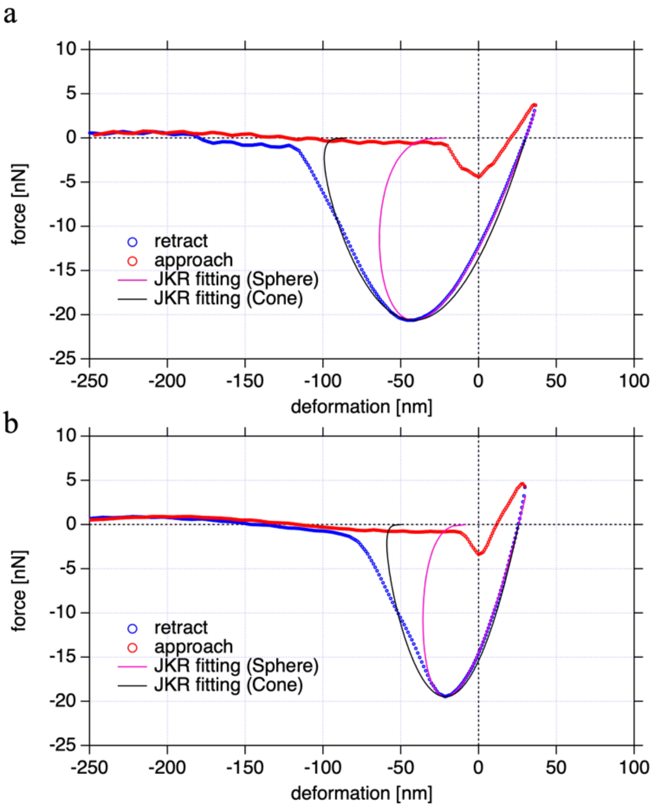

Figure S4. JKR model fitting of force-deformation curves. (a) rubber matrix region, (b) interface region.

The tip radius of the cantilever used in this experiment is about 15-20nm, and the deformation of the sample is about 10-40nm. The contact between the probe and the sample surface may be intermediate between the sphere assumption and the cone assumption. We compared the fitting curves of the two models, as shown in Figure S3. It can be seen that the curve assumed by the sphere fits the experimental curve better. Therefore, we chose the sphere model for calculation in this study.

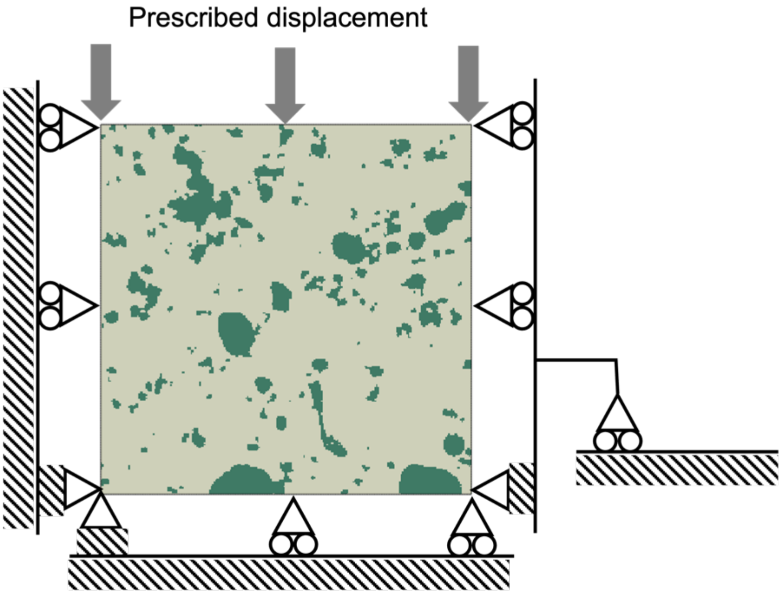

Figure S5. The boundary conditions in FEM simulations

As shown in the Fig. S5, each boundary of the FEM is constrained to hold a straight line, and uniform compressive deformation is given by prescribed displacement.
